# Ectopic transcription due to inherited histone methylation may interfere with the ongoing function of differentiated neurons

**DOI:** 10.1073/pnas.2513137122

**Published:** 2025-09-24

**Authors:** Juan D. Rodriguez, Monica N. Reeves, Sindy R. Chavez, Hsiao-Lin V. Wang, Jaely Z. Chavez, Rhea Rastogi, Liyang I. Sun, Mackenzie S. Roberson, Elicia A. Preston, Zaynab Massenburg, Kiani N. Cruz, Madhav S. Chadha, Emily J. Hill, Miguel L. Soares, Victor G. Corces, Brandon S. Carpenter, Karen L. Schmeichel, John I. Murray, David J. Katz

**Affiliations:** ^a^Department of Cell Biology, Emory University School of Medicine, Atlanta, GA 30322; ^b^Department of Human Genetics, Emory University School of Medicine, Atlanta, GA 30322; ^c^Department of Molecular and Cellular Biology, Kennesaw State University, Kennesaw, GA 30322; ^d^Department of Genetics, Perelman School of Medicine, University of Pennsylvania, Philadelphia, PA 19104; ^e^Department of Biology, Oglethorpe University, Brookhaven, GA 30319

**Keywords:** lineage, chromatin, *Caenorhabditis elegans*, LSD1, chemotaxis

## Abstract

How mutations in histone modifying enzymes lead to neurodevelopmental defects is unknown. To address this question, we took advantage of the invariant embryonic lineage and adult nervous system in *Caenorhabditis elegans* to investigate a double mutant between *spr-5/Lsd1/Kdm1a* (H3K4me1/2 demethylase) and *met-2/Setdb1/Kmt1e* (H3K9 methyltransferase). We demonstrate that *spr-5; met-2* double mutant worms have a severe chemotaxis defect caused by the ectopic expression of germline genes in somatic tissues. Despite this behavioral defect, we observe few embryonic lineage alterations and the normal complement of neurons. This raised the possibility that the abnormal chemotaxis behavior may be due to ongoing defects in terminally differentiated cells rather than alterations in development. Consistent with this possibility, we find that shutting off the ectopic expression of germline genes specifically in neurons rescues chemotaxis in *spr-5; met-2* mutants. Remarkably, shutting off the ectopic germline expression in *spr-5; met-2* adult worms, that had a chemotaxis defect earlier in development, is also sufficient to rescue chemotaxis behavior. These results suggest that ongoing inappropriate transcription in neurons can block normal behavior despite the chemotaxis neurons being intact.

## Significance

Inheritance of inappropriate chromatin in a double mutant between two histone modifying enzymes in *Caenorhabditis elegans* causes abnormal chemotaxis behavior in the progeny due to the widespread inappropriate expression of germline genes. We found that the behavior is abnormal despite there being no defects in neurodevelopment and that the abnormal behavior can be reverted by turning off the inappropriate germline transcription after the nervous system has fully formed. By showing that chromatin mutations can alter behavior without altering neurodevelopment, our work raises the possibility that human mutations in histone modifying enzymes may also not alter neurodevelopment. Therefore, it may be possible to reverse the altered behavior and intellectual disability observed in these patients by reversing transcription at any life stage.

Mutations in many chromatin regulators give rise to human neurodevelopmental disorders, characterized by behavioral abnormalities and intellectual disability (1). However, it remains unclear how defects in chromatin regulation give rise to neurodevelopmental defects. Therefore, to understand how defects in the regulation of histone modifying enzymes give rise to neurodevelopmental defects we investigated a double mutant between the H3K4me1/2 demethylase SPR-5 (referred to as LSD1/KDM1A in mammals) and the H3K9 methyltransferase MET-2 (referred to as SETDB1/KMT1E in mammals) in *Caenorhabditis elegans*.

H3K4 methylation is acquired cotranscriptionally via the COMPASS complex interacting with RNA polymerase and is associated with actively transcribed genes (2, 3). H3K4 methylation may function as an epigenetic memory, maintaining transcription over time or through mitotic cell divisions (4, 5). As a result, H3K4 methylation that is acquired during the production of gametes may have to be erased to prevent this epigenetic memory from being propagated across generations. In *C. elegans*, SPR-5 is required maternally to erase H3K4me2 and prevent it from being inherited transgenerationally (6). Without SPR-5, worms become increasingly sterile across generations (termed germline mortality) due to the transgenerational accumulation of H3K4me2 and the increasing expression of germline genes. Similarly, when LSD1/KDM1A is mutated maternally in mice, it results in embryonic lethality at the 2-cell stage (7, 8). This demonstrates that SPR-5/LSD1 has a conserved role in maternal epigenetic reprogramming.

H3K9 methylation is associated with repressed transcription (9). In *C. elegans*, loss of MET-2 also results in a germline mortality phenotype and *spr-5*; *met-2* double mutants have an exacerbated maternal effect sterility phenotype (10–12). This suggests that SPR-5 and MET-2 cooperate in maternal epigenetic reprogramming. The function of MET-2 in maternal reprogramming is conserved, as maternal loss of SETDB1/KMT1E in mice also results in early embryonic lethality (13).

During the production of the gametes in *C. elegans*, germline genes acquire the transcription-associated histone modification H3K36me3 via the H3K36 methyltransferase MET-1 (14). In the embryo, this H3K36 methylation is maintained by a transcription-independent H3K36 methyltransferase MES-4 at 176 critical germline genes (14, 15). For the remainder of the manuscript, we will refer to these genes as MES-4 targeted germline genes. At around the 60-cell stage, the germline blastomere P4 divides to give rise to the primordial germ cells, termed Z2 and Z3. Once the embryo hatches, the MES-4 targeted germline genes are expressed as Z2 and Z3 begin to proliferate and loss of MES-4 results in a maternal effect sterility phenotype (14, 15). Thus, it has been suggested that MES-4-dependent H3K36me3 may function as a type of bookmark to help respecify the germline in the subsequent generation. The transcription factor LSL-1 also plays a critical role in germline function. LSL-1 is first transcribed in P4, continues to be expressed during germline development in all four stages of larval development (L1 to L4), and remains on into the adult. Without LSL-1, the germline has several defects including defects in meiosis, germline apoptosis, and the production of almost no functional gametes (16).

Progeny of *spr-5; met-2* double mutants have a severe developmental delay at the L2 larval stage that is associated with the ectopic expression of MES-4 targeted germline genes in somatic tissues. In addition, progeny of *spr-5; met-2* mutants have ectopic H3K36me3 at MES-4 targeted genes in somatic tissues. When *mes-4* is knocked down by RNA interference in the progeny of *spr-5; met-2* mutants, the ectopic germline expression is eliminated and the L2 larval delay is rescued, suggesting that the severe developmental delay in the progeny of *spr-5; met-2* mutants is caused by the ectopic expression of MES-4 targeted germline genes (17). The ectopic maintenance of H3K36me3 in progeny of *spr-5; met-2* mutants is likely propagated by H3K4 methylation, because the developmental delay of *spr-5; met-2* mutants is also dependent on the H3K4 methyltransferase SET-2 (17). Similar to the progeny of *spr-5; met-2* mutants, loss of a NuRD complex component MEP-1 or DREAM complex component LIN-35 also results in the ectopic expression of germline genes and this ectopic expression is also dependent on MES-4 (18, 19). This suggests that these complexes may be functioning to reinforce SPR-5/MET-2 maternal reprogramming in somatic tissues. In the germline, the function of NuRD is antagonized by the germline transcription factor LSL-1 (16).

In *C. elegans*, the embryonic lineage is completely invariant. This means that every wild-type (N2) embryo undergoes the same pattern of cell division and cell migration (20). In order to facilitate study of the invariant lineage, the Waterston lab developed automated lineage tracing. The system utilizes a ubiquitously expressed histone-mCherry fusion protein to track cells via their nuclei with 3D time-lapse confocal imaging (21). *C. elegans* also have a simple completely mapped nervous system (22, 23). To enable the study of the nervous system, the Hobert lab developed the NeuroPAL worm in which each neuron expresses a combination of fluorescent molecules that, combined with cell position, allows the unique identification of all 302 neurons in the adult nervous system of *C. elegans* (24).

In the progeny of *spr-5; met-2* double mutants, H3K4me2 is inappropriately inherited from the previous generation and this results in the ectopic expression of MES-4 targeted germline genes (10, 17). Here, we take advantage of the invariant embryonic lineage and the simple completely mapped adult nervous system in *C. elegans* to understand how the inappropriate inheritance of chromatin and the ectopic expression of genes in the progeny of *spr-5; met-2* mutants effects individual cells and gives rise to abnormal behavior. To determine what embryonic cell types the MES-4 targeted germline genes are ectopically expressed in and how this may alter the embryonic lineage, we performed single-cell RNAseq and automated lineage tracing on the progeny of *spr-5; met-2* mutants. We find that MES-4 targeted germline genes begin to be ectopically expressed broadly in many embryonic lineages, except in the germline itself where MES-4 targeted germline genes may not be fully activated. Surprisingly, the ectopic expression of MES-4 targeted germline genes in somatic lineages results in few embryonic lineage defects and no gross morphological differences in the number or position of neurons. This raises the possibility that the ectopic expression of MES-4 germline genes does not primarily alter development but instead causes an ongoing defect in differentiated cells. While performing these experiments, we noticed that the progeny of *spr-5; met-2* mutants fail to move toward OP50 bacteria. Using chemotaxis assays, we found that the progeny of *spr-5; met-2* mutants have a chemotaxis defect that begins at the L2 stage and is dependent upon the expression of germline genes in neurons. This observation provided the opportunity to test whether the ectopic expression of germline genes may be actively interfering with the function of neurons by determining whether reverting the ectopic expression of these genes restores normal chemotaxis. Remarkably, we find that shutting off the ectopic expression of germline genes after the L2 stage rescues normal chemotaxis behavior. From these data, we conclude that the ectopic expression of germline genes in neurons can alter normal behavior despite the chemotaxis neurons being intact.

## Results

### Germline Genes Are Ectopically Expressed in the Somatic Lineages of *spr-5; met-2* Mutant Embryos.

**Fig. 1.**
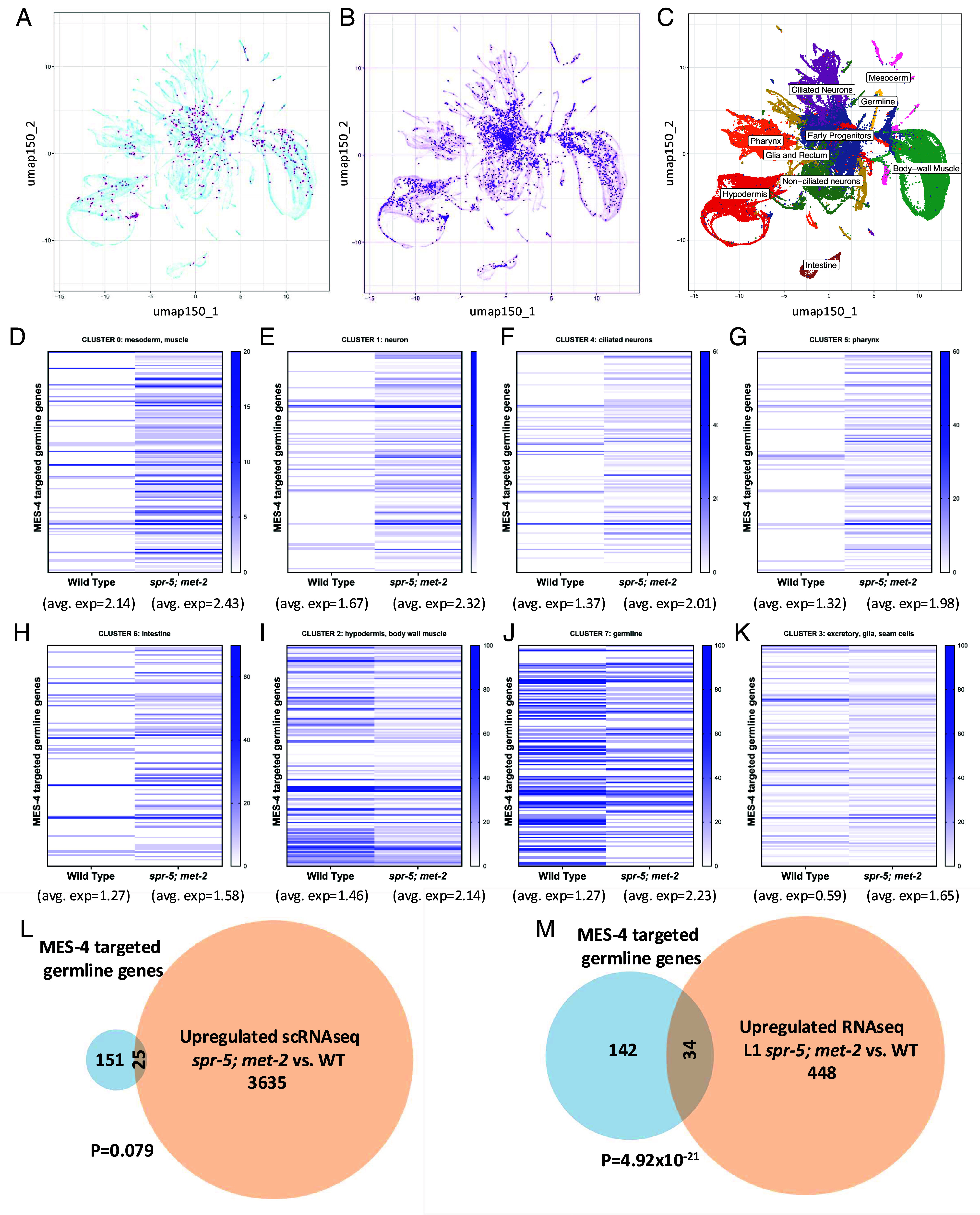
MES-4 targeted germline genes begin to be ectopically expressed during embryogenesis in *spr-5; met-2* mutants. (*A*–*C*) UMAP projection of all 219 Wild Type cells (N2) (*A*) and 686 *spr-5; met-2* mutant cells (*B*) from single-cell RNAseq integrated with the published (29) wild type (N2) single cell RNAseq data (purple dots show single-cell RNAseq compared to published data in lighter background). The cell type corresponding to each of the published single cell RNAseq clusters is shown in (*C*). (*D*–*K*) Heat maps corresponding to all 8 clusters obtained from unsupervised hierarchical clustering (from *SI Appendix*, Fig. S1*A*) showing the percentage of cells that express each of the 176 MES-4 targeted germline genes in Wild Type (N2) compared to *spr-5; met-2* mutants. The average gene expression for each cluster (shown below the cluster) represents the average of the normalized unique molecular identifier counts (corresponding to number of transcripts) across all 176 MES-4 targeted germline genes in any cell in which expression was detected. (*L* and *M*) Overlap between MES-4 targeted germline genes and total genes significantly upregulated in *spr-5; met-2* mutants in any of the 8 individual single-cell RNAseq clusters (*L*), or significantly upregulated in *spr-5; met-2* mutants at the L1 larval stage (*M*) from Carpenter et al. (17). Significance in (*L* and *M*) was determined by a hypergeometric test.

All of the experiments in this manuscript were performed on F1 progeny of homozygous *spr-5; met-2* double mutants to examine the effect of maternal loss of these enzymes. For simplicity, throughout the manuscript we will refer to these progeny simply as *spr-5; met-2* mutants. Previously we found in *spr-5; met-2* mutants that MES-4 targeted germline genes are ectopically expressed in the soma at the L1 stage (17). To determine how MES-4 targeted germline genes are misexpressed in the embryo at the single-cell level in *spr-5; met-2* mutants, we performed single-cell RNA sequencing on embryos enriched for the 100-200 cell stage (25). At this stage, early events of gastrulation are completed, zygotic transcription has fully initiated, and most cells have adopted broad tissue identities but are not yet terminally differentiating (26–28). We obtained sequences from 686 *spr-5; met-2* mutant cells and 219 wild-type cells. To ensure that the transcriptome of our wild-type cells match the published wild-type transcriptome, we integrated our small number of wild-type cells with the published wild-type *C. elegans* single cell atlas (29). To look for potential changes in transcriptomes, we also integrated our *spr-5; met-2* embryonic cells with the published wild-type *C. elegans* single cell atlas (29). Both wild-type and *spr-5; met-2* mutant cells mapped to cell states across the published wild-type dataset (Fig. 1 *A*–*C*). This indicates that broad cell classes are not disrupted and cell type specific marker genes are expressed in similar patterns in *spr-5; met-2* mutants. Nevertheless, using a 0.25 log2 fold change cutoff we identified 3,660 genes significantly up regulated and 1,679 genes significantly down regulated in *spr-5; met-2* mutants compared to Wild Type, most of which had relatively low fold changes. The larger number of up regulated genes compared to down regulated genes is consistent with SPR-5 and MET-2 acting as transcriptional repressors.

To investigate the single cell gene expression dataset further, we performed unsupervised hierarchical clustering of our stage-matched wild-type and *spr-5; met-2* mutant cells. Unsupervised clustering identified 8 individual clusters corresponding to major cell classes (germline, muscle, epidermis, etc) and these individual clusters are highly similar between wild-type and *spr-5; met-2* mutants (*SI Appendix*, Fig. S1 *A* and *B*). This confirms that cell type specific gene expression is not broadly changed overall in *spr-5; met-2* mutants compared to Wild Type.

To determine whether MES-4 targeted germline genes are ectopically expressed in different lineages, we also examined the gene expression (normalized unique identifier counts corresponding to number of transcripts) of all MES-4 targeted germline genes in each cluster. The average gene expression across all MES-4 targeted germline genes is higher in *spr-5; met-2* mutants compared to Wild Type (Fig. 1 *D*–*K*). We also determined the percentage of cells within each cluster that express each of the MES-4 targeted germline genes (Fig. 1 *D*–*K*). In 5 of the 7 somatic clusters (clusters 0,1,4,5,6) there is a large increase in the percentage of cells within the cluster that express MES-4 targeted germline genes (Fig. 1 *D*–*H*). Consistent with this, 25 of the 176 examined MES-4 targeted germline genes are significantly ectopically expressed in one of the 7 somatic clusters (Fig. 1*L* and Dataset S1). Together these results suggest that MES-4 targeted germline genes begin to be widely expressed in many somatic lineages in the embryo.

Despite the ectopic expression of MES-4 targeted germline genes in *spr-5; met-2* mutants overall, in the germline cluster (cluster 7) as well as the hypodermis and body wall muscle cluster (cluster 2) the percentage of cells within the cluster that express MES-4 targeted germline genes decreases in *spr-5; met-2* compared to Wild Type (Fig. 1 *I* and *J*). Consistent with this decrease in the percentage of cells expressing MES-4 targeted genes in the germline, there are only 20 MES-4 targeted germline genes significantly decreased in any cluster and 16 of them are in cells within the germline cluster (cluster 7) (Dataset S1). However, the decrease in the percentage of cells expressing MES-4 targeted genes in clusters 7 and 2 occurs despite there still being an increase in the average gene expression of the MES-4 targeted germline genes in these clusters (Fig. 1 *I* and *J*), as there is in all clusters (Fig. 1 *D*–*K*). Overall, the decrease in the expression of some MES-4 targeted germline genes in the germline cluster (cluster 7) is consistent with the possibility that many MES-4 germline genes fail to activate in the germline of *spr-5; met-2* mutants. It is not clear why MES-4 targeted germline genes are also expressed in fewer cells within cluster 2, but this effect appears to be driven by a higher percentage of these cells expressing MES-4 germline genes in Wild Type. In the excretory, glia, and seam somatic cell cluster (cluster 3), the percentage of cells within the cluster that express MES-4 targeted germline genes is unchanged between Wild Type and *spr-5; met-2* mutants (Fig. 1*K*). This raises the possibility that cells of this lineage may be resistant to the ectopic expression of MES-4 targeted germline genes, though it is possible that the lack of ectopic expression of MES-4 targeted germline genes in cluster 3 is simply due to low coverage in the scRNAseq dataset.

Single cell RNAseq is vulnerable to missing low levels of ectopic expression due to low numbers of RNAs in each cell. Therefore, to determine whether other MES-4 targeted genes that were not identified in the single cell RNAseq may also be ectopically expressed in *spr-5; met-2* mutants, we performed quantitative RT-PCR on 5 of the MES-4 targeted germline genes that were not significantly misexpressed in our single-cell dataset but were found to be significantly misexpressed at the L1 stage in *spr-5; met-2* mutants in our previous work (17). We found that 4 out of the 5 genes tested were significantly upregulated in 100-200 cell stage embryos with an average fold change of 3.5 (*SI Appendix*, Fig. S1*C*). Consistent with these results, we previously found by single molecule RNA fluorescence in situ hybridization that one of these genes, *htp-1,* is widely ectopically expressed in the somatic cells of the embryo (17). Finally, we also compared the ectopic expression of MES-4 targeted germline genes in our single-cell RNAseq dataset to our previously published *spr-5; met-2* RNAseq performed at the L1 larval stage. In contrast to our single cell dataset, where only 25 MES-4 targeted germline genes are significantly upregulated each only in a single cluster, in our previous L1 RNAseq dataset there were 34 MES-4 targeted genes that were significantly upregulated across the entire L1 larvae (Fig. 1*M*). This raised the possibility that MES-4 targeted germline genes may be increasingly ectopically expressed in larvae compared to embryos. To directly test this possibility, we performed quantitative RT-PCR on 8 of the significantly misregulated MES-4 targeted germline genes across development in *spr-5; met-2* mutants; at the 200-cell embryo stage, the comma stage of embryonic development and at the L1 larval stage. We found that the ectopic expression levels of these genes was highly variable, with some MES-4 targeted genes unchanged and some increasing across development, while others decreased across development and some that either increased then decreased, or decreased then increased across development (*SI Appendix*, Fig. S1*D*). This suggests that even if MES-4 targeted genes are becoming more consistently expressed in a wider number of cells across development, the overall levels of ectopic expression in the soma are not consistently increasing. Overall, our single cell RNAseq data suggest that inappropriate chromatin inherited from the previous generation in *spr-5; met-2* mutants is permissive for facilitating ectopic expression beginning widely during embryonic stages.

To determine whether the ectopic expression of germline genes in *spr-5; met-2* mutant embryos causes the ectopic production of germline proteins, we performed two experiments. First, we examined the ectopic production of the germline transcription factor LSL-1 using a GFP-tagged LSL-1 transgenic strain in *spr-5; met-2* mutants. At the 500-cell stage, LSL-1 protein is confined to the two germline blastomeres Z2 and Z3, as it is in Wild Type (*SI Appendix*, Fig. S2 *A*–*F*). But by the two-fold stage of embryogenesis, we detect ectopic LSL-1 protein in the soma of *spr-5; met-2* mutants (*SI Appendix*, Fig. S2 *G*–*L*). Second, we performed immunofluorescence with the OIC1D4 antibody that recognizes P granules. Compared to Wild Type 200-cell and comma stage embryos where P granules are confined to the two germline blastomeres Z2 and Z3 (*SI Appendix*, Fig. S3 *A*–*F*), we find ectopic P granules in *spr-5; met-2* mutants (*SI Appendix*, Fig. S3 *G*–*L*). Overall, these data confirm that the ectopic expression of germline genes in the soma of *spr-5; met-2* embryos results in the ectopic production of germline proteins there.

### *spr-5; met-2* Mutants Have a Delay in the Specification of the Germline, but No Defects in Somatic Embryonic Development.

**Fig. 2.**
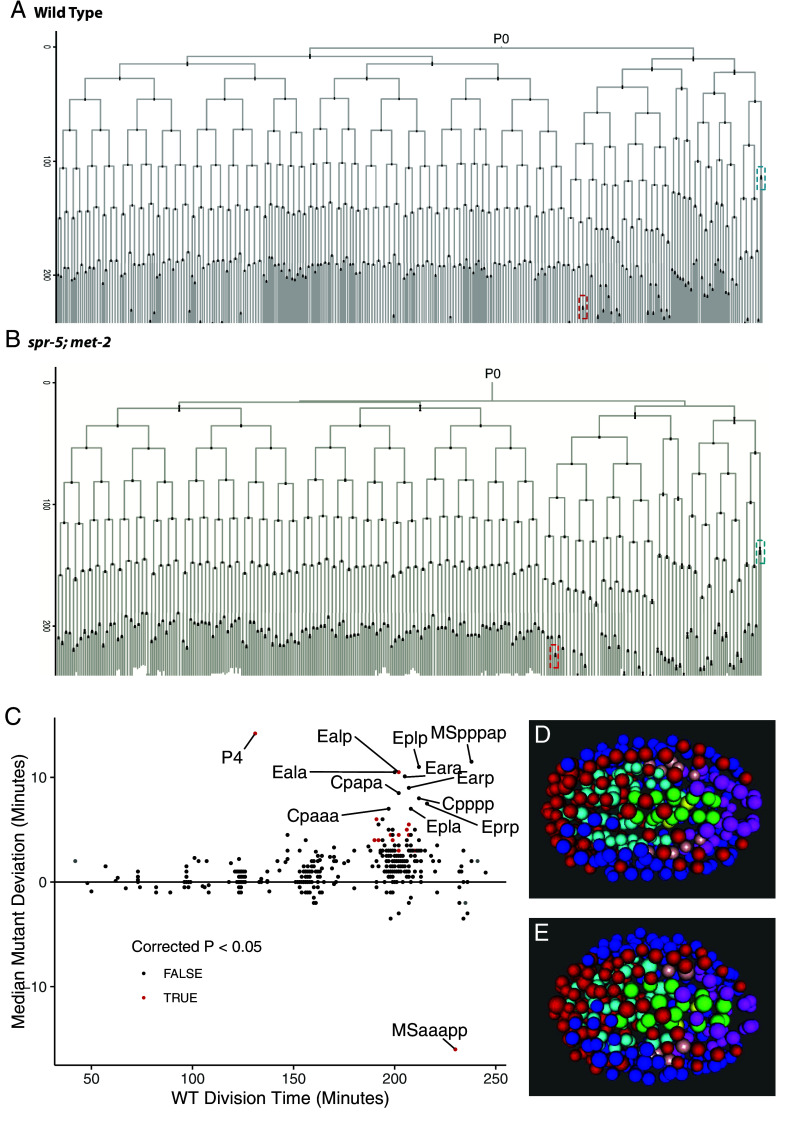
Comparison of the embryonic cell lineage in *spr-5; met-2* versus Wild Type. Wild Type (N2) (*A*) and *spr-5; met-2* (*B*) embryonic lineages through the 350-cell stage. The error bars indicate the SEM from 22 Wild Type (N2) and 8 *spr-5; met-2* lineages. The Y axis indicates minutes. The red dashed boxes indicate the earlier MSaaapp cell division and the blue dashed boxes indicate the later P4 cell division in *spr-5; met-2* compared to Wild Type (N2) (See *SI Appendix*, Fig. S4 for detail). (*C*) Plot of the median deviation in minutes of each cell in *spr-5; met-2* (N = 18) compared to Wild Type (N2) (N = 32) from all lineages collected through the 200 and 350 cell stages. Cells that significantly deviate from Wild Type (N2) (Bonferroni-corrected *P* < 0.05; 2 sample *t* test, unequal variance) are indicated in red (Datasets S1 and S2). (*D* and *E*) Average projection of Wild Type (N2) (*D*) and *spr-5; met-2* (*E*) embryos at the 350-cell stage (taken from Movie S1) indicating that all cells are in the correct position. Cells from major lineages are colored as Red:ABa, Blue:ABp, Cyan:MS, Green:E, Purple:C, Pink:D, and Yellow:P4.

To determine whether cell lineage specification is altered in *spr-5; met-2* mutants, we took advantage of the invariant embryonic lineage in *C. elegans*. By performing automated lineage tracing (30), we tracked the number of cell divisions, timing of cell divisions, cell position, and cell migration for all embryonic cells from the 2-cell stage through the 200-cell stage (~170 min) in 10 *spr-5; met-2* mutant embryos, and an additional 8 embryos through the 350-cell stage (~250 min) (Datasets S1 and S2). Surprisingly, we found no major defects in any somatic lineages deriving from either the AB or P1 blastomeres through the 200-cell stage and only a few significant defects through the 350-cell stage (Fig. 2 *A*–*C* and Datasets S2 and S3). The cell division of the pharyngeal intestinal valve cell MSaaapp occurs significantly earlier in *spr-5; met-2* mutants compared to Wild Type (Fig. 2 *A*–*C* and *SI Appendix*, Fig. S4 *A* and *B*). In addition, we observe modest delays in the myo-epidermal C lineage and intestinal E lineage in *spr-5; met-2* mutants compared to Wild Type. These defects are less severe than defects seen in mutants for transcription factors involved in fate specification (31–33) and only a few of these cells are significantly delayed (Fig. 2*C*). Furthermore, the division times of the myo-epidermal C lineage and intestinal E lineage are typically more variable than others even in Wild Type animals (33). Despite the slight lineage defects that we observe in *spr-5; met-2* mutants, all embryonic cells are present in the correct place through the 350-cell stage (Fig. 2 *D* and *E* and Movie S1 *A* and *B*). Overall, these data suggest that somatic development is almost completely normal in *spr-5; met-2* mutant embryos.

In contrast, we identified a consistent delay in the germline lineage, with the germline blastomere P4 dividing to give rise to the two primordial germ cells, Z2 and Z3, significantly later in *spr-5; met-2* mutants compared to Wild Type (Fig. 2 *A*–*C* and *SI Appendix*, Fig. S4 *C* and *D*). The delay in the cell division of the germline blastomere P4 occurs despite there being no delay in the cell D, the somatic sister of P4 (*SI Appendix*, Fig. S4*D*). The P4 delay correlates with the failure to fully express MES-4 targeted germline genes in the germline cluster (cluster 7) (Fig. 1*J*), raising the possibility that the delayed division of P4 could be due to the failure to properly activate germline transcription.

### *spr-5; met-2* Mutants Have a Severe Defect in Chemotaxis Toward OP50 Bacteria.

**Fig. 3.**
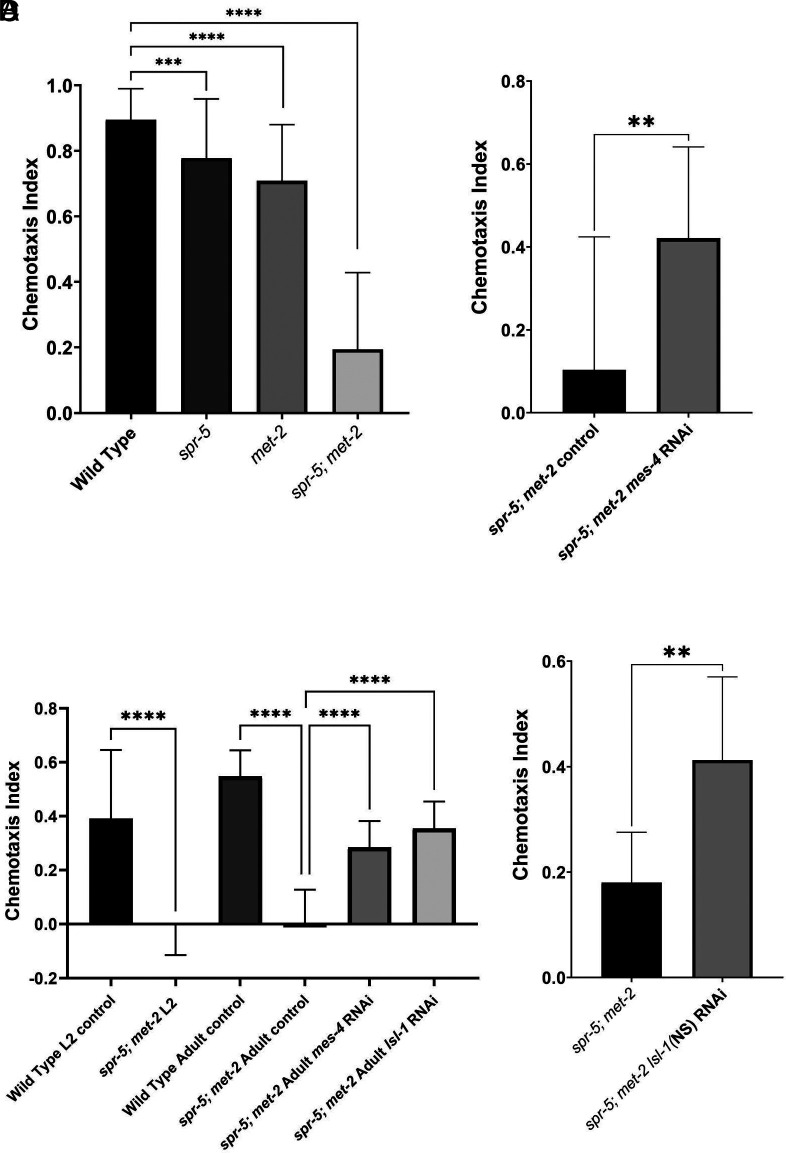
Blocking the ectopic transcription of MES-4 targeted germline genes in L2 *spr-5; met-2* mutants that already have a chemotaxis defect restores normal chemotaxis. (*A*) The chemotaxis index of Wild Type (N2) (N = 1,450 worms from 36 assays) (Movie S2*A*), *spr-5* (N = 2,352 from 55), *met-2* (N = 1,751 from 51), and *spr-5; met-2* (N = 443 from 19) adults. (*B*) The chemotaxis index of *spr-5; met-2* mutant adults is rescued when *spr-5; met-2* mutants are developed in the presence of *mes-4* RNAi (N = 603 from 15) compared to control (L4440) RNAi (N = 287 from 14). (*C*) The chemotaxis index of L2 Wild Type (N2) (N = 681 from 23), L2 *spr-5; met-2* double mutants (N = 2,950 from 37) is rescued by shifting on to *mes-4* (N = 763 from 12) (Movie S2*C*) or *lsl-1* (N = 776 from 14) RNAi (Movie S2*E*) at the L2 stage and then assaying chemotaxis in the resulting adults. These resulting adults, which are the same worms that previously had a chemotaxis defect at the L2 stage, are rescued to levels that are similar to Wild Type (N2) (N = 1,003 from 9) (Movie S2*D*) under these conditions. In contrast, control (L4440) RNAi does not rescue (N = 603 from 14) (Movie S2*B*). Worms were assayed as adults ~5 d after initiating RNAi at the L2 stage. (*D*) The chemotaxis index of *spr-5; met-2* double mutants (N = 590 from 6) is rescued by *lsl-1* RNAi driven by a pan-neuronal promoter [*lsl-1*(*NS*)] (N = 1,018 from 21). The chemotaxis in *B*–*D* was performed with OP50 bacteria after carrying out RNAi using HT115 bacteria. The error bars represent the SEM. Significance was calculated in (*A*–*D*) by the unpaired *t* test. **** ≤0.0001, *** ≤0.001, ** ≤0.01. The raw data are included as Dataset S4.

While maintaining *spr-5; met-2* mutants and performing these experiments, we noticed that *spr-5; met-2* mutants fail to preferentially move toward the OP50 *Escherichia coli* food source. To quantify this behavior defect, we performed a chemotaxis assay and calculated the chemotaxis index (CI). A chemotaxis index of 1.0 indicates that all of the worms are located in the quadrants with the OP50 *E. coli* food source, while a chemotaxis index of 0.0 indicates that the worms are randomly distributed throughout the experimental plate. Compared to Wild Type worms which have a chemotaxis index of 0.9 (Movie S2*A*), *spr-5* and *met-2* single mutants have a small but significant defect in chemotaxis, with chemotaxis indices of ~0.7. In contrast, *spr-5; met-2* mutants have a severe chemotaxis index defect of 0.2, confirming the synergistic interaction between *spr-5* and *met-2* (Fig. 3*A* and Movie S2*B*). The chemotaxis defect of *spr-5; met-2* mutants is not specific to OP50, as we also detect a significant chemotaxis defect when benzaldehyde or diacetyl is used as the odorant (*SI Appendix*, Fig. S5).

The *spr-5; met-2* chemotaxis defect is observed at both the L2 larvae and adult stages compared to Wild Type controls (Fig. 3). As has previously been described, the chemotaxis index of L2 Wild Type animals is slightly lower than adult animals (Fig. 3) (34). To determine whether the *spr-5; met-2* chemotaxis defect could be due to a lack of motility, we also tracked the total distance covered during the 1 h chemotaxis assay and the distance covered from minute 2 to 6 of the chemotaxis assay, before Wild Type reaches the food. Neither of these distances moved was significantly different between Wild Type and *spr-5; met-2*, suggesting that the chemotaxis defect is not simply due to a lack of motility (*SI Appendix*, Fig. S6). Taken together, these results demonstrate that *spr-5; met-2* chemotaxis mutants fail to preferentially move toward the OP50 *E. coli* food source, despite embryonic development being almost completely normal in these mutants.

### The *spr-5; met-2* Chemotaxis Defect is Dependent Upon the Ectopic Expression of MES-4 Targeted Germline Genes.

Previously we demonstrated that a severe larval (L1/L2 stage) developmental delay also observed in *spr-5; met-2* mutants can be rescued by knockdown of the H3K36 methyltransferase MES-4 (17). This suggested that the developmental delay is dependent upon the ectopic expression of MES-4 targeted germline genes. To determine whether the chemotaxis defect of *spr-5; met-2* mutants is also dependent upon the ectopic expression of MES-4 germline genes, we measured chemotaxis in *spr-5; met-2* mutants which developed in the presence of *mes-4* RNA interference (RNAi). RNAi of *mes-4* in *spr-5; met-2* mutants significantly rescued the chemotaxis defect, suggesting that the chemotaxis defect is also dependent upon the ectopic transcription of MES-4 targeted germline genes (Fig. 3*B*).

### Zygotic Knockdown of Ectopic MES-4 Targeted Germline Gene Transcription in L2 *spr-5; met-2* Mutants Restored Normal Chemotaxis in These Same Animals as Adults.

The lack of embryonic developmental defects in *spr-5; met-2* mutants raised the possibility that the severe chemotaxis defect in these mutants could be due to the ongoing ectopic expression of MES-4 targeted germline genes. If this is the case, it might be possible to shut off the ectopic expression of the MES-4 targeted germline genes and restore normal chemotaxis. To test this possibility, we took L2 *spr-5; met-2* larvae which already have a severe chemotaxis defect and shut off the ectopic expression of MES-4 targeted germline genes by shifting to *mes-4* RNAi at the L2 stage and then assaying chemotaxis in the resulting adults. Importantly, because *spr-5; met-2* double mutants have a severe developmental delay which is not rescued when *mes-4* RNAi is initiated at the L2 stage, it takes ~5 d for them to develop from L2 larvae to adults. This gives the RNAi plenty of time to knock down the ectopic transcription of the MES-4 targeted germline genes in somatic tissues. It is also possible that RNAi works better in the soma of *spr-5; met-2* double mutants because of the ectopic expression of germline RNAi machinery (17). Of note, in these experiments (and the previous MES-4 RNAi experiments in Fig. 3*B*) the RNAi was performed with HT115 bacteria rather than OP50, prior to assaying chemotaxis toward the OP50 *E. coli* food source. This results in an overall reduction in the chemotaxis index (Fig. 3*C*). For example, under these conditions the chemotaxis index of wild-type animals is 0.55 (or 0.4 in the previous MES-4 RNAi experiments in Fig. 3*B*) rather than 0.9, and the chemotaxis index of *spr-5; met-2* double mutants is 0.0 (or 0.1 in the previous MES-4 RNAi experiments in Fig. 3*B*) rather than 0.2.

Remarkably, we find that shutting off the ectopic germline expression of the MES-4 targeted germline genes after the L2 stage results in a significant rescue of the chemotaxis index from 0.0 to 0.28 in these same animals at the adult stage (Fig. 3*C* and Movie S2 *B*–*D*). MES-4 is required to propagate the ectopic transcription of MES-4 targeted germline genes to somatic tissues but may not be completely required to continually maintain the ectopic transcription of MES-4 targeted germline genes in somatic cells. Therefore, we also tried to shut off the ectopic expression of germline genes by performing RNAi against *lsl-1.* LSL-1 is a germline transcription factor that is actively required for the transcription of many germline expressed genes, including some MES-4 targeted germline genes (16). We found that knockdown of LSL-1 after the L2 stage in *spr-5; met-2* mutants also results in the rescue of the chemotaxis index in *spr-5; met-2* mutants from 0.0 to 0.36 (compared to 0.28 with *mes-4* RNAi) (Fig. 3*C* and Movie S2*E*). As with the MES-4 RNAi experiments, these chemotaxis experiments were performed in the same adult animals that were previously defective at the L2 stage. Our finding that the chemotaxis behavior is reversible by shutting off the ectopic expression of germline genes is even more remarkable considering that the nervous system is already formed in L2 larvae when we begin the RNAi. Taken together these results suggest that the ongoing ectopic transcription of MES-4 targeted germline genes can block normal chemotaxis behavior despite the nervous system being intact enough to be rescued.

### Knockdown of Germline Genes in Neurons Rescues the *spr-5; met-2* Chemotaxis Defect.

To determine whether it is the ectopic expression of germline genes specifically in neurons that interferes with normal chemotaxis behavior in *spr-5; met-2* mutants, we also performed RNAi against *lsl-1* in neurons. To do this we generated transgenic worms in which dsRNA against *lsl-1* is driven by the *unc-119* pan-neuronal promoter (35). RNAi of *lsl-1* in *unc-119* expressing cells is sufficient to significantly rescue the chemotaxis defect in *spr-5; met-2* mutants from 0.18 to 0.41 (Fig. 3*D*). This suggests that it is the ectopic expression of germline genes in neurons that is causing the chemotaxis defect in *spr-5; met-2* mutants.

### The Adult Nervous System is Completely Present and Properly Located in *spr-5; met-2* Double Mutants.

**Fig. 4.**
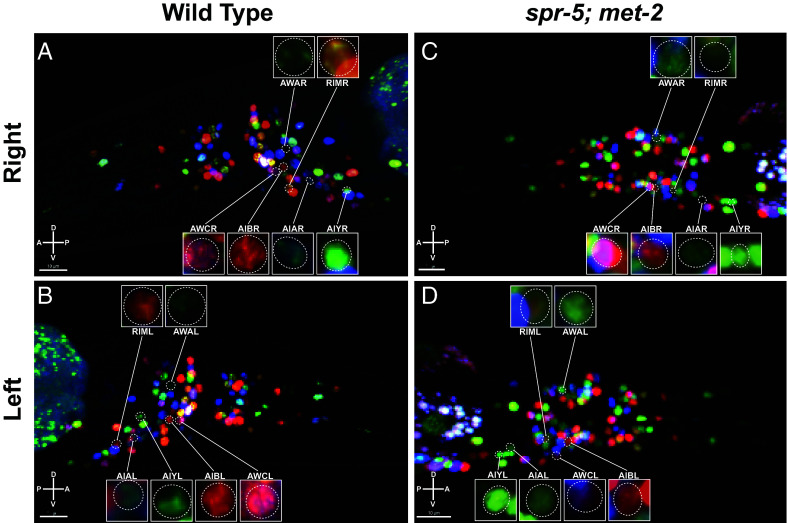
The nervous system is entirely present and properly located in *spr-5; met-2* mutants. Example projections of Wild Type (N2) (*A* and *B*) and *spr-5; met-2* mutant (*C* and *D*) adult heads, both the *Right* sides (*A* and *C*) and *Left* sides (*B* and *D*) of the heads are shown. The *Left* and *Right* pairs of sensory neurons (AWA and AWC) and interneurons (AIA, AIB, and AIY), as well as the *Left* and *Right* pair of motor neurons (RIM) that are known to specifically function in chemotaxis toward the OP50 *E. coli* food source are labelled with zoomed-in insets (3D rotation in Movie S3 *A* and *B*).

Our finding that knockdown of *mes-4* or *lsl-1* by RNAi rescues normal chemotaxis in *spr-5; met-2* double mutants suggests that the chemotaxis neurons are intact in these animals. However, to confirm that these and other neurons are present in *spr-5; met-2* double mutants, we took advantage of the invariant adult nervous system in *C. elegans*, which can be imaged using the NeuroPAL system (24). To verify that the NeuroPAL system is working, we first located all of the neurons in the different parts (head, tail, etc) of the wild-type NeuroPAL strain (Fig. 4 *A*, *B*, and *E* and *SI Appendix*, Fig. S7). Then we compared these different parts of the worm between *spr-5; met-2* mutants and Wild Type. This analysis demonstrated that there are no differences in number of neurons or general position of these neurons between *spr-5; met-2* mutants and Wild Type (Fig. 4 *C*–*E* and *SI Appendix*, Fig. S7). This includes the key neurons that are known to specifically function in chemotaxis toward the OP50 *E. coli* food source: the left/right pairs of olfactory sensory neurons AWA and AWC, the left/right pairs of interneurons AIA, AIB, and AIY, as well as the left/right pair of motor neurons RIM (Movie S3 *A* and *B*) (36–42). Thus, in *spr-5; met-2* mutants the ongoing ectopic transcription of MES-4 targeted germline genes can block normal chemotaxis behavior, despite the nervous system being fully present, in a way that is reversible by shutting off the ectopic transcription. It should be noted that occasionally the position or color of individual neurons varied slightly between *spr-5; met-2* and Wild Type. For example, we observed slightly different coloring in RIMR (Fig. 4 *A* vs. *C*). But this level of slight variation is also observed between different individual Wild Type NeuroPAL worms (24).

## Discussion

*spr-5; met-2* double mutants inappropriately inherit chromatin from the previous generation and ectopically express germline genes in somatic tissues. This provided the unique opportunity to determine how the ectopic expression of genes resulting from inappropriately inherited histone methylation influences behavior. Previously, we found that *spr-5; met-2* mutants have a severe L2 developmental delay that is associated with the ectopic expression of MES-4 targeted germline genes. Knock down of *mes-4* by RNAi eliminates the ectopic expression of MES-4 targeted germline genes and rescues the L2 developmental delay, suggesting that the developmental delay is dependent upon the ectopic expression of MES-4 targeted germline genes (17). Here, we found that *spr-5; met-2* mutants have a severe defect in chemotaxis toward OP50 bacteria at both the L2 larval stage and in adults. There is also a significant chemotaxis defect in both *spr-5* and *met-2* single mutants, but the chemotaxis defect is much more severe in *spr-5; met-2* double mutants, confirming the synergistic interaction between *spr-5* and *met-2*. In addition, we find that the severe chemotaxis defect in *spr-5; met-2* mutants is dependent upon MES-4, suggesting that this phenotype is also dependent upon the ectopic expression of MES-4 targeted germline genes.

The ectopic expression of MES-4 targeted germline genes is observed equally in most somatic clusters. This is consistent with a model in which the ectopic chromatin is inherited fairly uniformly across embryonic lineages. But we do not observe the ectopic expression of the MES-4 targeted germline genes in the excretory, glia, and seam somatic cell cluster (cluster 3), so some lineages may not inherit the ectopic chromatin or may be resistant to the ectopic expression of these germline genes. However, we cannot rule out the possibility that lack of ectopic expression of the MES-4 targeted germline genes in the excretory, glia, and seam somatic cell cluster (cluster 3) is simply due to low coverage in these cells. Despite the ectopic expression of MES-4 germline genes, unsupervised clustering analysis of our *spr-5; met-2* single-cell RNAseq data demonstrated that these mutants are very similar to Wild Type. This suggests that the ectopic transcription does not broadly interfere with cell specification in the embryo. Consistent with this possibility, our automated lineage tracing of *spr-5; met-2* mutants demonstrated that there are very few defects in the embryonic lineage. Using NeuroPAL imaging, we also observed that *spr-5; met-2* mutants have the same complement of uniquely identifiable neurons as Wild Type, including all the neurons that are known to be required for normal chemotaxis toward the OP50 *E. coli* food source. The lack of embryonic lineage and adult nervous system defects is surprising because they occur despite there being a severe developmental delay and impaired chemotaxis beginning at the L2 stage.

The lack of defects caused by the inappropriate somatic expression of germline genes is consistent with two possible models. One possibility is that embryonic development is resistant to the MES-4 targeted germline genes that are significantly ectopically expressed in the embryo. In this scenario, development may overcome the ectopic expression. Alternatively, it is possible that the critical MES-4 targeted germline genes that cause the L2 developmental delay and chemotaxis defect are not yet misexpressed at a sufficient level to cause these defects in the embryo. Our finding that the overall levels of ectopic expression of MES-4 targeted genes do not consistently increase across development from the 200-cell embryo stage to the comma and L1 larval stage is more consistent with the possibility that embryonic development is resistant to the ectopic expression of MES-4 targeted germline genes. We also detect that the ectopic expression of germline genes results in the ectopic production of germline proteins in the embryo, so the lack of embryonic defects in *spr-5; met-2* mutants is not due to the failure of the ectopically expressed germline genes to be translated. However, since it is not clear which ectopically expressed MES-4 targeted gene or genes causes the chemotaxis defect, it remains possible that chemotaxis defect is caused by the subset of MES-4 targeted germline genes that do increase across development. In this case, it is possible that lack of embryonic defects could be caused by the insufficient ectopic expression earlier in development. Regardless, the lack of somatic embryonic lineage defects combined with the presence of the full complement of neurons raised the possibility that the developmental delay and chemotaxis defects in *spr-5; met-2* mutants are not due to a failure to properly specify a specific lineage during embryogenesis.

Although we do not observe any significant somatic defects in the embryonic lineage of *spr-5; met-2* mutants, we detect a delay in duration of the germline blastomere P4 before dividing to give rise to the primordial germ cells Z2 and Z3 in *spr-5; met-2* mutants compared to Wild Type. This delay occurs despite there being no corresponding delay in the P4 sister cell D, arguing that the P4 delay is highly specific to the germline lineage. While it remains unclear why there is a delay in the P4 cell division, our single-cell data demonstrate that MES-4 targeted genes fail to be fully expressed in the germline cluster (7). Therefore, it is possible that the failure to activate the transcription of the MES-4 germline genes causes a delay in the P4 cell division. The failure to fully activate germline transcription in Z2 and Z3 may also account for why *spr-5; met-2* mutants are completely sterile (10, 11). Previously, it has been shown that the target of SPR-5, H3K4me2, is specifically lost during the P4 cell division to generate Z2 and Z3 (43). Thus, it is possible that an increase in H3K4me2 in *spr-5; met-2* mutants results in a failure to erase H3K4me2 in Z2 and Z3. This in turn could lead to the delay in the P4 cell division, but this possibility remains to be examined. Alternatively, it is possible that the germline delay is a secondary consequence of the somatic defects. This possibility would be consistent with previous data suggesting that the nervous system can communicate to the germline (44–46).

The lack of somatic embryonic lineage defects in *spr-5; met-2* mutants raised the possibility that the chemotaxis defect is due to the ongoing ectopic expression of germline genes. If this were the case, we would expect that eliminating the ectopic expression in worms that already have a chemotaxis defect would eliminate the chemotaxis defect. To test this possibility, we performed *mes-4* RNAi beginning at the L2 stage in *spr-5; met-2* mutants to block the ectopic expression of MES-4 targeted germline genes, when these mutants already have a chemotaxis defect. Importantly, at the L2 larval stage *C. elegans* already have an intact nervous system. Remarkably, we find that knock down of *mes-4* beginning at the L2 stage significantly rescues chemotaxis behavior in the same animals that were previously defective at the L2 stage. MES-4 is thought to function in propagating the inappropriate expression of MES-4 targeted germline genes in somatic tissues and may not be completely required to maintain the ectopic expression of these genes. Therefore, we also tried to block the ectopic expression of germline genes by performing RNA interference against the germline transcription factor LSL-1. LSL-1 is required for the transcription of many germline genes, including many MES-4 targeted germline genes. Knock down of *lsl-1* beginning at the L2 stage in *spr-5; met-2* mutants also rescued chemotaxis behavior. Taken together these results suggest that the ectopic expression of MES-4 targeted germline genes actively interferes with normal chemotaxis behavior despite the chemotaxis neurons being intact.

To determine whether it is the ectopic expression of germline genes specifically in neurons that interferes with normal chemotaxis behavior, we also performed RNAi of *lsl-1* in *spr-5; met-2* neurons. RNAi of germline genes from a pan-neuronal promoter rescues the chemotaxis defect of *spr-5; met-2* mutants, suggesting that it is the ectopic expression of germline genes in neurons that is interfering with normal chemotaxis behavior. Consistent with this possibility, we find that *spr-5; met-2* mutants move the same amount as Wild Type worms, suggesting that the chemotaxis defect of *spr-5; met-2* mutants is not due to a defect in motility.

Our data suggest that the ectopic expression of MES-4 targeted germline genes actively interferes with normal chemotaxis behavior despite the chemotaxis neurons being intact. However, it remains unclear how the ectopic expression of germline genes actively interferes with normal chemotaxis behavior. One possibility is that some normal component of neuronal function is blocked at the transcription level. For example, the expression of synaptic proteins or a chemoreceptor could be actively inhibited in *spr-5; met-2* mutants. An alternative possibility, that is not mutually exclusive, is that some aspect of germline function actively interferes with neuronal function. For example, activation of meiosis genes could cause inappropriate chromosomal condensation in neurons. Regardless, our finding that ectopic transcription actively interferes with intact chemotaxis neurons may have implications for neurodevelopmental diseases caused by mutations in histone modifying enzymes, such as Kabuki Syndrome (47) and phenotypically similar LSD1 patients (48, 49). Based on our results, it is intriguing to speculate that the intellectual disability and/or altered behavior observed in these patients could be due to an ongoing defect in a properly formed nervous system. Even though these patients have de novo mutations that were either inherited from one of their parents or mutated in the very early embryo, it is possible that the inappropriately inherited chromatin results in a defect that only manifests itself later in development because the transcription factors that are required to interact with the permissive chromatin are only activated in certain differentiated cell types, such as neurons. Regardless of the etiology, if the nervous system defects in these patients are due to the ongoing ectopic expression of genes, it may be possible to rescue these defects by turning off the ectopic transcription. Consistent with this possibility, it has been recently shown that behavioral defects in two mouse models of epigenetic syndromes can be rescued at the adult stage. Loss of the DNA methylation binding protein MeCP2 that causes Rhett Syndrome can be rescued by reexpressing MeCP2 in adult mice (50–52). In addition, heterozygous loss of the H3K4 methyltransferase *Kmt2d* that causes Kabuki Syndrome can be partially rescued in adult mice with a histone deacetylase inhibitor (53), but the reason that these models can be rescued at adult stages remains unknown. Our data provide insight into these results.

## Materials and Methods

### Strains.

All *C. elegans* strains were cultured at 20˚C on 60 mm nematode growth media (NGM) agar plates with OP50 bacteria grown in Luria Broth (LB). Strains used were N2: Wild Type (Bristol isolate); the *C. elegans spr-5* (*by101*)(*I*) strain was provided by R. Baumeister (Albert Ludwig University of Freiburg, Germany); the MT13293: *met-2* (*n4256*)(*III*) *strain* was provided by R. Horvitz (Massachusetts Institute of Technology, MA). *spr-5*(*b101*)/*tmC27*[*unc-75*(*tmls1239*)] I*; met-2*(*n4256*)]/*qC1 qIs26* [*lag-2::GFP + pRF4 rol-6*(*su1006*)] III strain was created for maintenance as a double heterozygote. The automated cell lineage strain is *spr-5*(*b101*)/*tmC27*[*unc-75*(*tmls1239*)] I; JIM113: ujIs113 [Ppie-1::H2B::mCherry, unc-119(+); Pnhr-2::HIS-24::mCherry, unc-119(+) II; *met-2*(*n4256*)]/*qC1 qIs26* [*lag-2::GFP + pRF4 rol-6*(*su1006*)] III. The NeuroPAL strain otIs669 V (Wild Type) was obtained from *Caenorhabditis* Genetics Center (CGC) and crossed into *spr-5; met-2* mutants. The neuron specific *lsl-1* RNAi strain (*Punc-119-lsl-1 sense chain, Punc-119-lsl-1 anti-sense chain, Pmyo-3::mCherry*) was obtained from Suny Biotech. The *unc-119* promoter sequence is included as Dataset S5. *lsl-1* RNAi sequence was the same as the Ahringer library (54).

### Single-Worm Genotyping.

Single animals were picked into 10 to 12 μL of lysis buffer [50 mM KCl, 10 mM Tris-HCl (pH 8.3), 2.5 mM MgCl_2_, 0.45% NP-40, 0.45% Tween-20, 0.01% gelatin] containing 1 µg/µL final concentration of proteinase K and incubated at −80 °C overnight followed by 60 °C for 1 h and then 95 °C for 15 min. PCRs were performed with AmpliTaq Gold (Invitrogen) according to the manufacturer’s protocol and reactions were resolved on agarose gels. The following genotyping primers were used: spr-5 (*by101*)I: fwd: AAACACGTGGCTCCATGAAT, rev(wt):GAGGTTTTGAGGGGTTCCAT, rev(mut):CTTGAAACAGACTTGAACATCAAAGATCGG; met-2 (n4256): fwd(wt):GTCACATCACCTGCATCAGC, rev(wt):ATTTCATTACGGC TGCCAAC, fwd(mut):ATTCGAAAAATGGACCGTTG, rev(mut):TCTATTCCCAGGAGCCAATG.

### Quantitative PCR.

Total RNA was isolated using TRIzol reagent (Invitrogen) from synchronized embryos at room temperature. cDNA synthesis and qPCR were carried out as previously described (10). mRNA was quantified by real-time PCR, using iO SYBR Green Supermix (BioRad). The following primers were used: *htp-1* (ATTCGGAGGACAGTGACACAA and GTGCTTTCTCGAGAGACTCAGTTATATC) *cpb-1* (GTGCTGATTGATTGGCCTCG and CCGTTACAGCGCGTGAACCG); *rmh-1* (TGTAGTCATTATGCCAAGTATCTGC and ATCTGTTACTCGTATCTGTAGTAGCC); *ftr-1* (TCCGCTCACTTCGAATACGG and TACCATCGCGATTGTGAGC); *fbxa-101* (TATCGAAGACAAGCTCGCCG and TGCGAACGGAAATCCAATCG); *ama*-1 (TACCTACACTCCAAGTCCATCG and CGATGTTGGAGAGTACTGAG); *wago-1* (CTCTGGGTGAACTAACGACTA and ACGCGCTTCTGATTGAGATC); *glh-4* (ACTATTTCGATGCATAGCTACCA and CCACGAGCACACACATTTGA); *ego-1* (TCAATATGCGTCTTCTGGTTTCT and CACATCTTGTCCCGTTACGT); *nxf-2* (TGTATCCGGACAGTGAGAGC and GAATTGTGGCAGGAAGTCTCG); *ani-2* (ATGCACAAATCAGTCGGTCG and GGCTAGCACAACTCGTCTTT); *tofu-4* (ACCATTTACCCATCTCGTCTTC and TCCTTCATCAGTTTGGAGTCTCT); *clec-91* (TCAGTCTTGTCATGGATCGGT and GGTTTGATGAGCCAGTTGATCT); *glh-2* (TGCCGAACGTGGACTTGATA and CTTCCAGCGTTTCCAACTCG). Each mRNA expression was normalized to *ama-1* control. Fold enrichment was calculated as mutant/Wild Type.

### RNA Interference.

*E. coli* HT115 was transformed with a vector expressing dsRNA of *mes-4*, *lsl-1*, L4440 or *ama-1* obtained from the Ahringer library (Source BioScience). RNAi bacteria and empty vector control were grown at 37 °C and seeded on RNAi plates (standard NGM plates containing ampicillin at 100 mg/ml, tetracycline at 5 mg/ml and isopropylthiogalactoside (IPTG) at 0.4 mM). RNAi plates were left at room temperature to induce for at least 48 h.

### Chemotaxis Assay.

The experimental 60 mm NGM plate was divided into four equal quadrants with 5µL of control LB/vehicle in two quadrants (C1 and C2) and 5 µL of 200X concentrated fresh overnight cultures of *E. coli* OP50 in the other two quadrants (E1 and E2). Worms were collected and rinsed 3 times with M9 buffer (22 mM KH_2_PO_4_, 42 mM Na_2_HPO_4_, 86 mM NaCl, and 1 mM MgSO_4_). A total of ~50 worms per plate were placed into the center of the experimental plate. The plates were then incubated at 20 °C for 1 h. Worms that moved to any of the quadrants after the incubation time were recorded. The Chemotaxis Index was calculated as follows; (E1 + E2) – (C1 + C2)/Total worms moved.

### Worm Tracking.

Video was recorded of Wild Type and *spr-5; met-2* young adult worms during a chemotaxis assay as described above. The number of worms used for each video was <10 to enable tracking. LoliTrack V5 (Loligo Systems) was used to analyze the distance moved for each individual worm in order to calculate the average distance moved.

### Lineage Tracing.

Cell lineage tracing was carried out as previously described using a Zeiss LSM 510 confocal microscope with some minor changes (30). While using the 20 µm beads, we noticed that the embryo was moving out of focus. To rectify this, an NGM plate was used as a platform to hold the embryo. This alternative approach is described in detail (55). Additional embryos were imaged using a Leica Stellaris confocal microscope. Cell lineages were traced using StarryNite, curated using AceTree, and Cell positions and division times compared to a wild-type compendium as in (21, 30, 32, 33, 56–58).

### Single-Cell RNA Sequencing and Data Analysis.

Single-cell isolation was performed according to Packer et al. (29) with minor modifications. For the 100 to 200 cell stage, only one enzyme (Chitinase) was used for embryo eggshell disruption and single cells dissociation (55). Single-cell RNA sequencing was performed using the 10X Genomics single-cell capturing system. Single cells were isolated from embryos produced by approximately 1,000 hand-selected *spr-5; met-2* mutant mother worms per strain. 1,000 to 3,000 cells were loaded on the 10X Genomics Chromium Controller. Single-cell cDNA libraries were prepared using the Chromium Next GEM Single Cell 3’ LT Reagent Kits v3.1 (Dual Index). Libraries were sequenced by FSU College of Medicine Translational Science Laboratory (Florida State University, FL). After the 10X QC, Wild Type had 219 estimated cells, 62,491 mean reads per cell and 1,067 median genes per cell. *spr-5; met-2* had 686 cells, 20,763 mean reads per cell and 526 median genes per cell. The number of cells was limited by the need to hand-pick *spr-5; met-2* double mutant mothers prior to embryo collection. Sequencing: 28 million paired-end of ~150 bp paired-end reads (Illumina NextSeq 600). The Cell Ranger Software Suite 3.0.2 (10X Genomics) was used for the alignment of the single-cell RNA-seq output reads and generation of feature, barcode, and matrices. Seurat analysis was used for unsupervised hierarchical cluster formation.

### NeuroPAL.

10-15 young adult stage worms were paralyzed with 10 mM levamisole and mounted on a 1.5% agarose gel pad. The coverslip was sealed with nail polish to reduce evaporation and warping of the gel pad during lengthy imaging. Worms were imaged on a Nikon A1R HD25 inverted confocal microscope with optical configurations and imaging channels from the NeuroPAL manual “Configuring Your Microscope for NeuroPAL v3,” obtained from Hobert Lab website https://www.hobertlab.org/neuropal/ (24). Images were taken at 60X with 3 channels: CyOFP, pseudocolored green, mNeptune2.5, pseudocolored red, mTagBFP2, pseudocolored blue, using a Z-stack with 30 to 50 steps of 0.8 μm. Images were taken without the TagRFP-T pan-neuronal marker, pseudocolored in white. A minimum of 3 images were taken of each worm: head, midbody, and tail. The resulting images were adjusted for brightness/contrast and channels were merged into a composite using Fiji/ImageJ (59). 3D images were obtained using Imaris software version 9.8.0 as described in (24, 60). Imaris 3D images were used to count the number of neurons in each worm and annotate the chemotactic circuit neurons. Neurons were identified using the “NeuroPAL Reference Manual v1” and “Using NeuroPAL for ID v1” manuals obtained from Hobert Lab website.

### LSL-1 GFP and P-Granule Immunofluorescence of Embryos.

Mixed staged embryos were isolated by washing off plates with embryos laid overnight by gravid adult hermaphrodites followed by bleaching to remove adults and larvae. Embryos were permeabilized on poly-L-lysine coated slides using the freeze-crack method and immediately fixed in methanol for 10 min then in acetone for 5 min as previously described (Duerr, 2013). The slides were then washed once with 1× PBST (phosphate buffer saline with 0.1% Tween-20) then blocked for 1 h in Antibody Buffer (1× PBST with 0.5% bovine serum albumin and 0.01% sodium azide). Primary antibody staining was conducted overnight at room temperature using a rabbit polyclonal anti-GFP antibody (ab6556, Abcam) at a 1:600 dilution to detect the *LSL-1*[syb3772(*LSL-1::gfp::ha::6xhis*)](*V*) allele or OIC1D4 (P granule) antibody (DSHB) at 1:2 dilution. After three washes with 1× PBST, secondary antibody staining was performed for 1 h at room temperature using an Alexa Fluor 488-conjugated goat anti-rabbit antibody (A11034, Invitrogen) at a 1:500 dilution. Following incubation with secondary antibody, slides were washed twice with 1× PBST and once with 1× PBST containing 200 ng/ml DAPI. After three washes with 1× PBST, slides were mounted in Vectashield mounting medium and imaged immediately using a 63x objective on a Zeiss LSM 900 Confocal microscope imaging system for LSL-1 or using a 60x objective on a Leica epifluorescence scope for P granules. ImageJ maximum projection was used to project z-stack images to a single plane for LSL-1.

## Supplementary Material

Appendix 01 (PDF)

Dataset S01 (XLSX)

Dataset S02 (XLSX)

Dataset S03 (XLSX)

Dataset S04 (XLSX)

Dataset S05 (PDF)

Movie S1. WT average projection (related to Fig. 3). *spr-5; met-2* average projection (related to Fig. 3)

Movie S2. WT chemotaxis on OP50 bacteria (related to Fig. 3). *spr-5; met-2* chemotaxis on HT115 bacteria (related to Fig. 3). *spr-5; met-2* on *mes-4* RNAi chemotaxis (related to Fig. 3). WT chemotaxis on HT115 bacteria (related to Fig. 3). *spr-5; met-2* on *lsl-1* RNAi chemotaxis (related to Fig. 3).

Movie S3. WT NeuroPAL rotation (related to Fig. 4). *spr-5; met-2* NeuroPAL rotation (related to Fig. 4)

## Data Availability

Single cell RNAseq dataset data have been deposited in Gene Expression Omnibus (GSE272897) (25). All study data are included in the article and/or supporting information.
