# Supplementary material for: Ectopic transcription due to inherited histone methylation may interfere with the ongoing function of differentiated neurons
Source: Proc Natl Acad Sci U S A. 2025 Sep 24;122(39):e2513137122. doi: 10.1073/pnas.2513137122 (PMC12501177; doi:10.1073/pnas.2513137122)
Supplement: Supplementary file 1 — Appendix 01 (PDF) [file pnas.2513137122.sapp.pdf]

PNAS

Supporting Information for
Ectopic transcription due to inherited histone methylation may interfere with the ongoing function of differentiated neurons

Juan D. Rodriguez[1*], Monica N. Reeves[1*], Sindy R. Chavez[1], Hsiao-Lin V. Wang[2], Jaely Z. Chavez[3], Rhea Rastogi[1], Liyang I. Sun[1], Mackenzie S. Roberson[1], Elicia A Preston[5], Zaynab Massenburg[3], Kiani N. Cruz[1], Madhav S. Chadha[1], Emily J. Hill[2], Miguel L. Soares[1], Victor G. Corces[2], Brandon S. Carpenter[3], Karen L. Schmeichel[4], John I. Murray[5], David J. Katz[1#]

[1]Department of Cell Biology, Emory University School of Medicine, Atlanta GA 30322, USA.
[2]Department of Human Genetics, Emory University School of Medicine, Atlanta GA 30322, USA.
[3]Department of Molecular and Cellular Biology, Kennesaw State University, Kennesaw, GA 30322, USA.
[4]Department of Biology, Oglethorpe University, Brookhaven GA 30319, USA.
[5]Department of Genetics, Perelman School of Medicine, University of Pennsylvania, Philadelphia PA 19104, USA
*co-first authors
#Corresponding author

David J. Katz
Email: djkatz@emory.edu

This PDF file includes:

Figures S1 to S7

Other supporting materials for this manuscript include the following:

Movies S1 to S3
Datasets S1 to S5

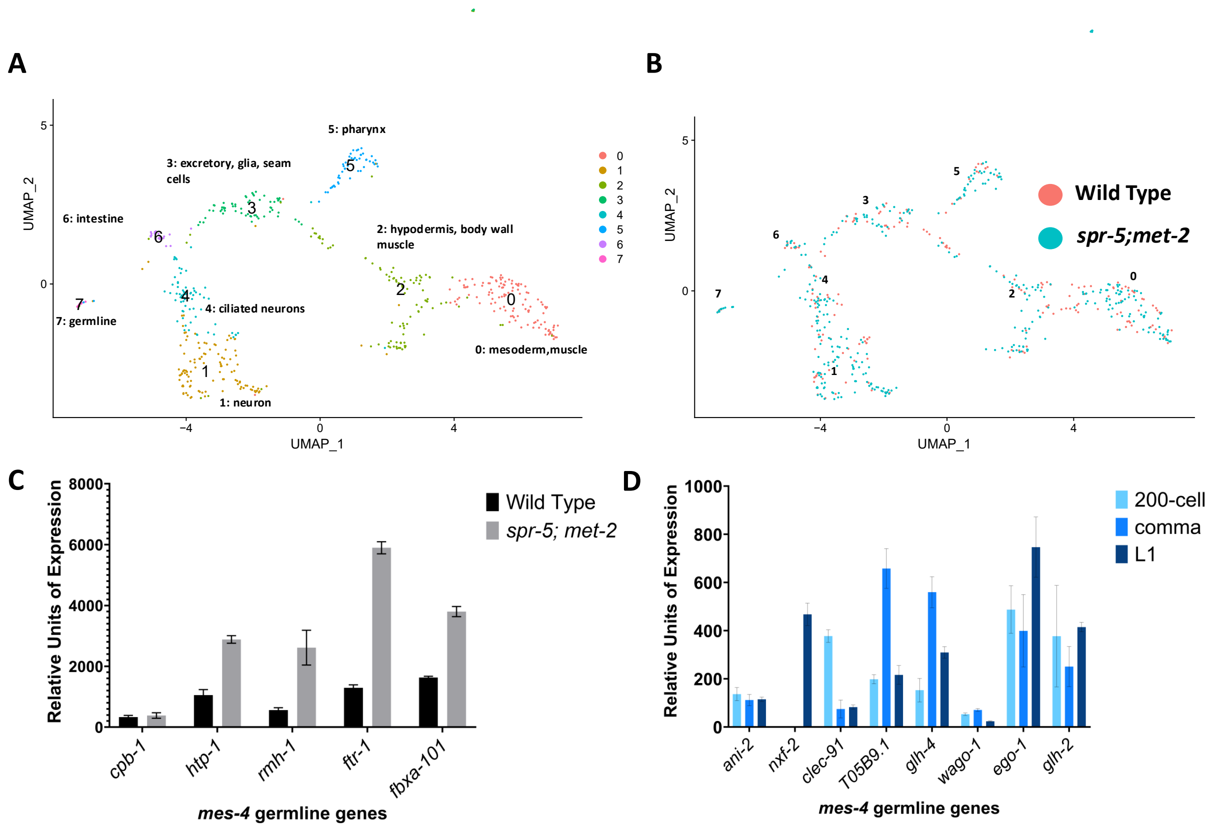

Fig. S1. Germline genes are ectopically expressed, but overall gene expression is largely unchanged during embryogenesis in *spr-5; met-2* mutants.
Unsupervised hierarchical clustering of single cell RNAseq data forms 8 clusters (**A**) and these clusters are the same between Wild Type (N2) (pink) and *spr-5; met-2* mutants (aqua) (**B**). (**C,D**) Quantitative RT-PCR data showing the relative gene expression of 5 MES-4 targeted germline genes not significantly misexpressed in our single-cell dataset but found to be significantly misexpressed at the L1 stage in *spr-5; met-2* mutants in our previous work (**C**) (17) and 8 MES-4 targeted germline genes most significantly misexpressed in our single-cell dataset at the 200-cell, comma and L1 larval stages (**D**). The data were normalized against the expression of *ama-1* from embryos at the 100-200 cell stage (**C**) and corresponding stages (**D**). The error bars (**C**,**D**) represent the S.E.M of 3 technical replicates from ≥200 pooled embryos or worms.

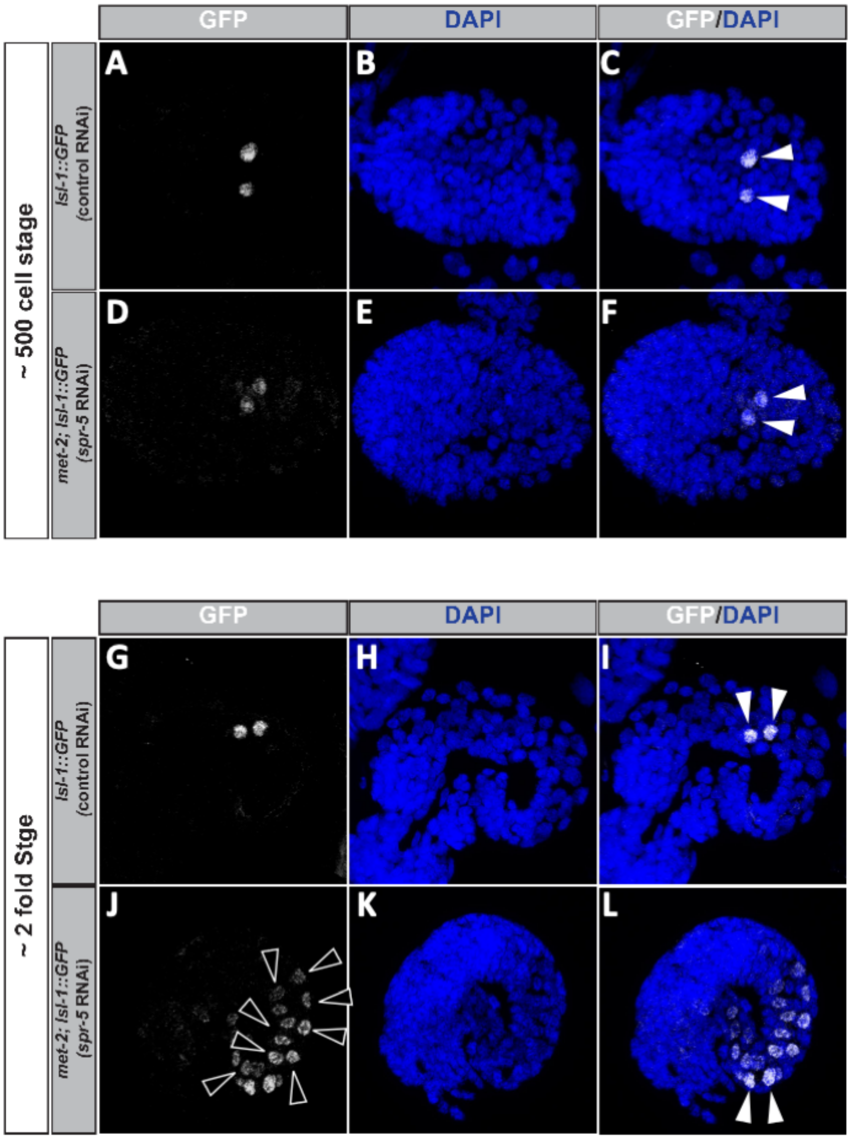

Fig. S2. Without SPR-5 and MET-2, LSL-1 is ectopically expressed in the soma. (A-L) Representative images of of *lsl-1::GFP* embryos from hermaphrodites fed control RNAi (A-F) and *met-2; lsl-1::GFP* embryos from hermaphrodites fed *spr-5* RNAi (G-L) at approximately the 500-cell (A-C, G-I) 2-fold stage (D-F, J-L) stages. DAPI was used to stain nuclear chromatin. White arrows indicate the primordial germ cells Z2 and Z3. Black arrows indicate ectopic expression of LSL-1 in somatic cells.

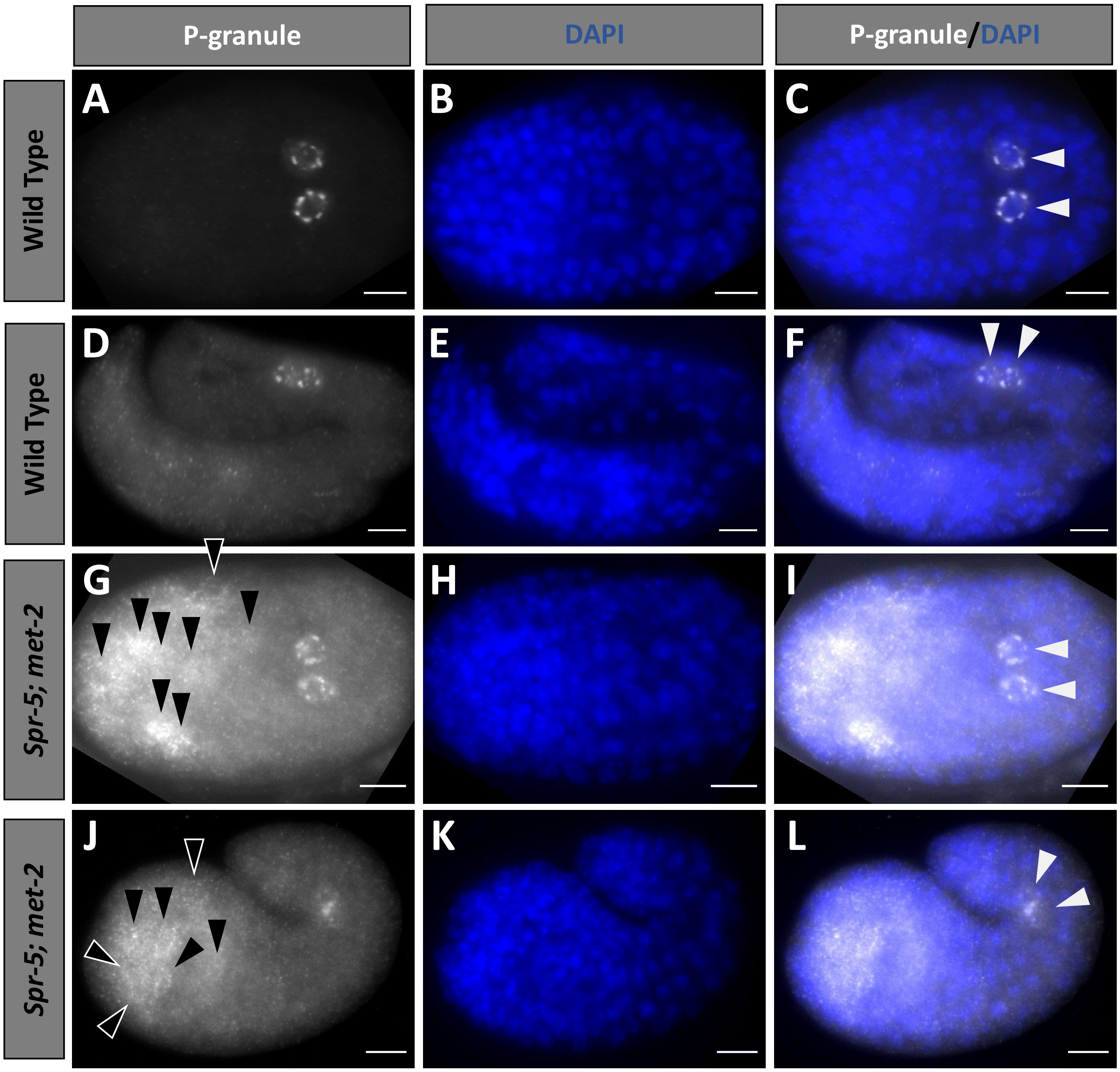

Fig. S3. Without SPR-5 and MET-2, P granules are ectopically expressed in the soma. Representative images of ectopic P granules in Wild Type (N2) (A-F) versus spr-5; met-2 mutants (G-L) at the ~200-cell (A,B,C,G,H,I) and comma stage (D,E,F,J,K,L) of embryogenesis. DAPI was used to stain nuclear chromatin. White arrows indicate the primordial germ cells Z2 and Z3. Black arrows indicate ectopic P granules in somatic cells.

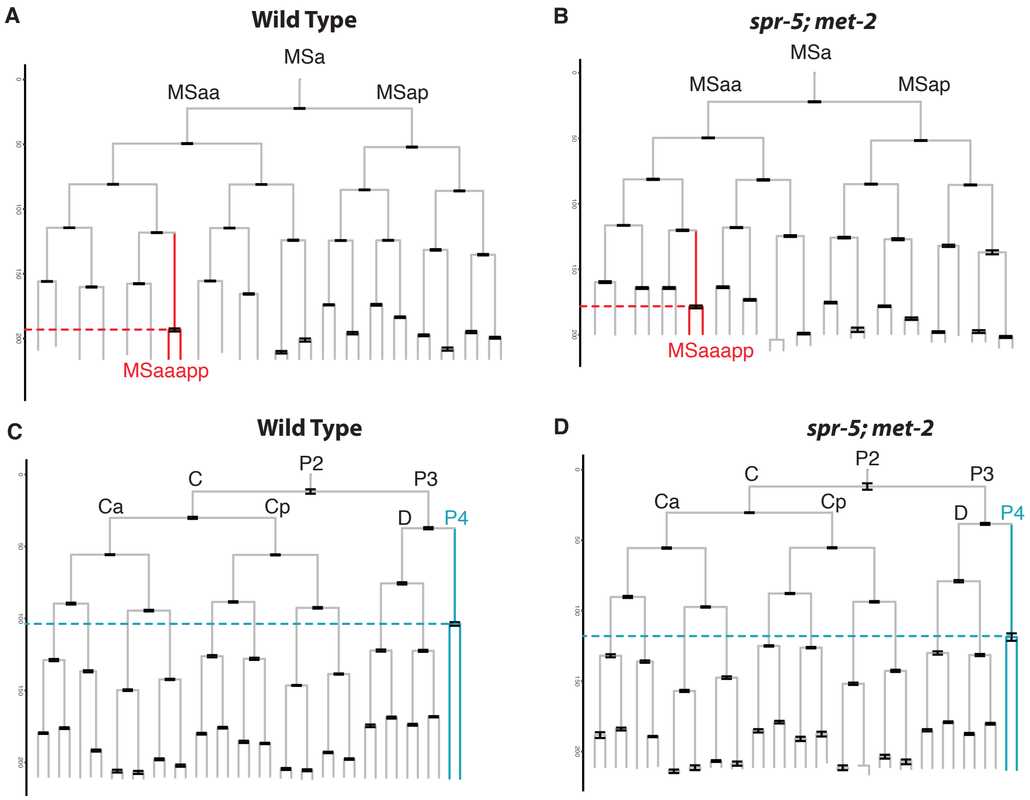

Fig. S4. **Comparison of the embryonic cell lineage in *spr-5; met-2* versus Wild Type.** Wild Type (N2) (**A,C**) and *spr-5; met-2* (**B,D**) embryonic lineages highlighting the earlier MSaaapp cell division (red dashed boxes in Fig. 1A,B) and the later P4 cell division (blue dashed boxes in Fig. 1A,B) in *spr-5; met-2* compared to Wild Type (N2). The error bars indicate the SEM from 22 Wild Type (N2) and 8 *spr-5; met-2* lineages.

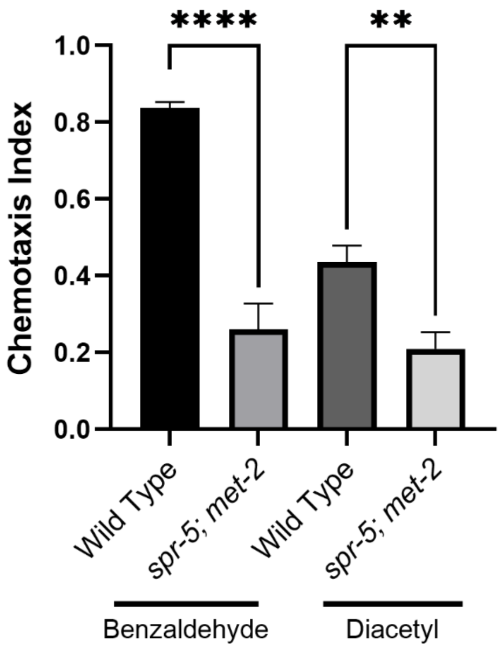

Fig. S5 *spr-5; met-2* mutants are defective in chemotaxis towards benzaldehyde and diacetyl. The chemotaxis index of Wild Type (N2) (N=633 worms from 8 assays) versus *spr-5; met-2* mutants (N=490 from 8) towards benzaldehyde, and Wild Type (N2) (N=893 from 8) versus *spr-5; met-2* mutants (N=541 from 8) towards diacetyl. The chemotaxis was performed on adult worms. Significance was calculated in by unpaired t-test. ****≤0.0001, **≤0.01. The raw data are included as Dataset S4.

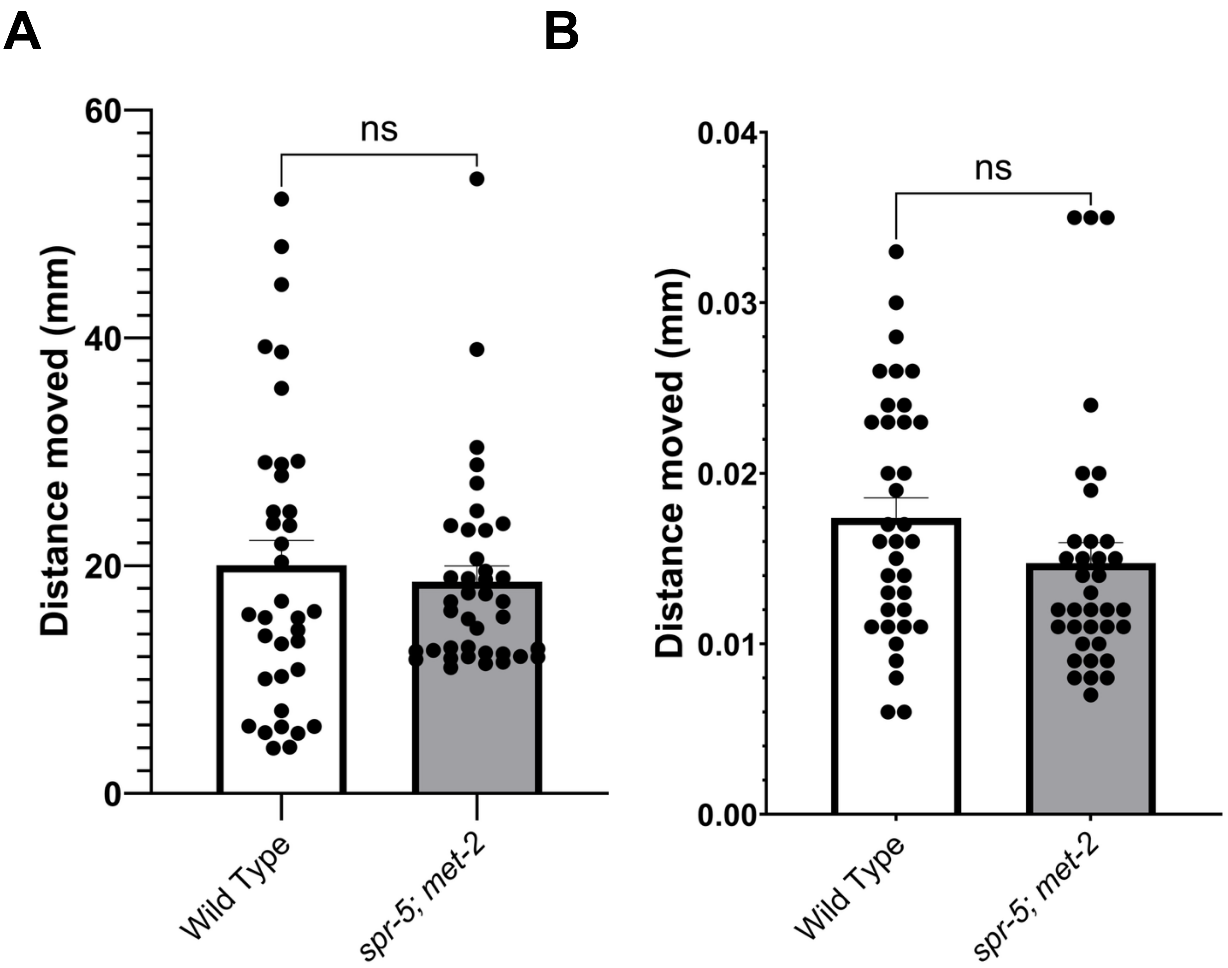

Fig. S6. The movement of *spr-5; met-2* mutants does not differ significantly from Wild Type during the chemotaxis assay. (**A**) The total distance travelled by Wild Type (N2) versus *spr-5; met-2* mutants is not significantly different during the 1hr chemotaxis assay. **(B)** The distance moved by Wild Type between minutes 2-6 of the chemotaxis assay, before Wild Type worms reach the food, is also not significantly different. Wild Type (N=36 from 10 assays) and *spr-5; met-2* mutants (N=36 from 10 assays).

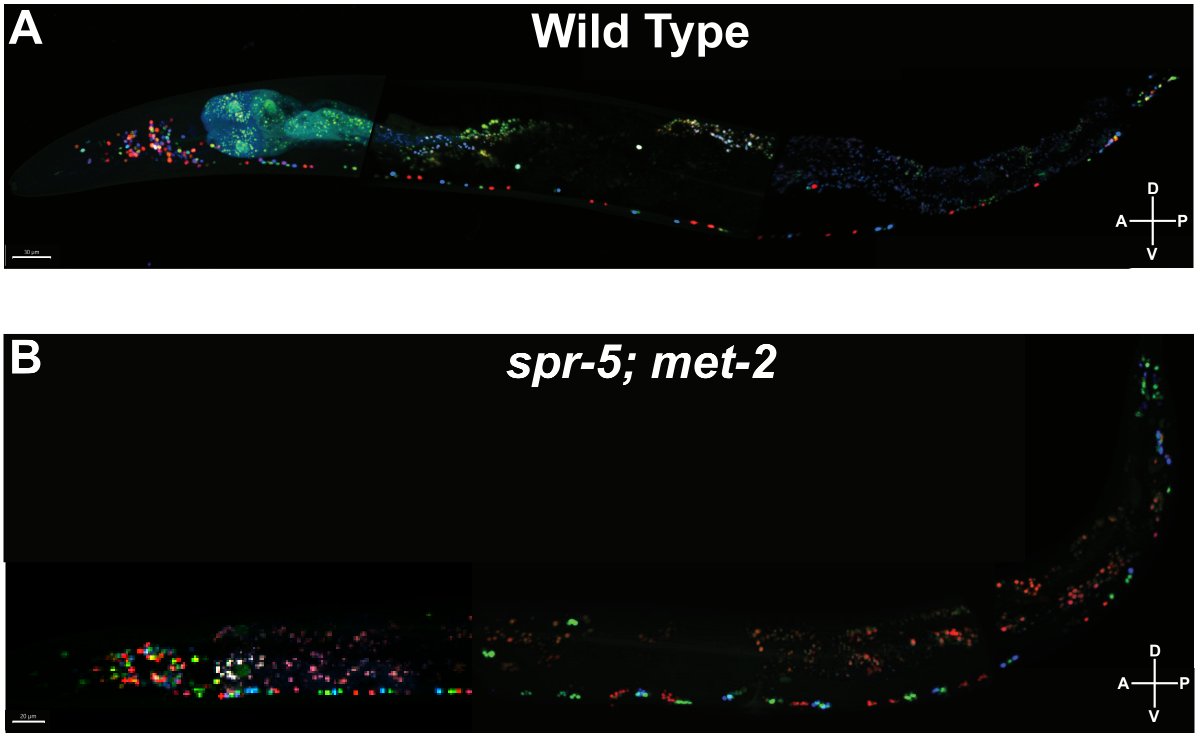

Fig. S7. The nervous system is entirely present and properly located in *spr-5; met-2* mutants with impaired chemotaxis.

Example of Wild Type (N2) (**A**) and *spr-5; met-2* (**B**) adult worms indicating that all 302 uniquely identified neurons are present in the correct position. The anterior(A)/posterior(P) and dorsal(D)/ventral (V) axes are indicated.

Supplementary Information

Dataset S1 list of MES-4 targeted germline genes up regulated in the soma or down regulated in the germline (related to Fig. 1)

Dataset S2 lineage tracing rate- corrected rate cycles (related to Fig. 2)

Dataset S3 lineage tracing- specific rates (related to Fig. 2)

Dataset S4 table of individual chemotaxis assays (related to Fig. 3)

Dataset S5. The *unc-119* promoter sequence used to drive *lsl-1* RNAi in neurons

Video S1A WT average projection (related to Fig. 3)

Video S1B *spr-5; met-2* average projection (related to Fig. 3)

Video S2A WT chemotaxis on OP50 bacteria (related to Fig. 3)

Video S2B *spr-5; met-2* chemotaxis on HT115 bacteria (related to Fig. 3)

Video S2C *spr-5; met-2* on *mes-4* RNAi chemotaxis (related to Fig. 3)

Video S2D WT chemotaxis on HT115 bacteria (related to Fig. 3)

Video S2E *spr-5; met-2* on *lsl-1* RNAi chemotaxis (related to Fig. 3)

Video S3A WT NeuroPAL rotation (related to Fig. 4)

Video S3B *spr-5; met-2* NeuroPAL rotation (related to Fig. 4)
