# Supplementary material for: Ectopic transcription due to inherited histone methylation may interfere with the ongoing function of differentiated neurons
Source: Proc Natl Acad Sci U S A. 2025 Sep 24;122(39):e2513137122. doi: 10.1073/pnas.2513137122 (PMC12501177; doi:10.1073/pnas.2513137122)
Supplement: Supplementary file 6 — Dataset S05 (PDF) [file pnas.2513137122.sd05.pdf]

Dataset S5- The unc-119 promoter sequence that was used for the neuron specific RNAi strain

atgttttggaaaatgtgtcaagtctctcaaaattcagtttttaaaccactcctatagtcctatagtcctatagttacccatgaaatccttatat
attactgtaaaatgtttcaaaaaccattggcaaattgccagaactgaaaatttccggcaaattggggaaccggcaaattgccaatttgctg
aatttgccggaaaaacggtaattgccgaaagtttttgacacgaaaatggcaaattgtggttttaaaattttttttttggaaatttcagaatttca
attttaatcggcaaaactgtaggcatcctaagaatgttcctacatctattttgaaaagtaagcgaattctatgaaaatgtctaaagaaaatgg
ggaaacaatttcaaaaaggcacagtttcaagtgtttccgaattatactaaatccctctaaaaacttccggcaaattgatatccgtaaaaga
gcaaatccgcatttttgccgaaaattaaaatttccgacaaatcggcaaaccggcaatttggcgaaatttgccggaaggcaattgccgccc
acccctgttccagaggttcaaactggtagcaaagctcaaaatttctcaaattctccaattttttttgaattttggcagtgtaccaaaatgaca
ttcagtcatattggtttattatagatttatttagataaaatcctaaatgattctacctttaaagatgcccactttaaaagtaatgactcaaacttc
aaattgctctaagattctattgaattaccatcttttcctctcattttctctcactgtctatttcatcacaaattcatccctctctccctctctttctc
tctccctctctctctttctctttgctcatcatctgtcattttgtccgttcctctctctgcgccctcagcgttccccacactctctcgcttctcttttc
ctagacgtcttctttttcatcttcttcagccctttttcgccattttccatctctgtcaatcattacggacgacccccattaatttttggatgtgct
ctgcgagcaagagcacacggaactcatttatttttgttggaatttctttttcattttagttagttaaaaggttattcagtgcaaaaattgatttttt
gctcttttggtacattagaggggtttgaaagttttccataaaaagggtttccggagtaaaaaatctaaaaatttttacaaacatttccaaaaat
tttgggaacatatcttttcaaaaactcaaaaaaaaacgcaaacatcataaatttaatcaattaattggatgcgttcaaatattttaaaaatat
ttttgaattgcatatttctttactcataaaaaaattattaatgtttaagtaaaaaaaatatttcaggattttaaacaaaaactgtaaatcccata
aaacagtgttattttttagttatttttagtaattatttgtgaattaaaaacacattttttttcaaaatattccttgaataaataaaattgaaagtgaa
ttcaaaatatttcaccaattttacaaaatttgaaaatttgaaaaaatattttctaccaaattgtgctcaaaaaatattattaagttcccaggaa
atttatgatgaaaacttggaatatttttggtaaaattttctcaaaaaattgaaagttgtcaaaaacacactaacactatacctcgcggcatag
aaaaaactggtggccgaatttttaaaccaatttaaattaaatatatttttgcactttgtacaaaaaaaatttgaagttcccaggaaattcatac
ggaaatattttccagaacttttataatttttattttaattctcaaaatttacaataaaaacttggaaacctgaaatttgcttaaagaataaccct
atcccccacggcctagaaaatactggtggccgaatttttccgcagccacaccacctctaatctccagagcactctccaaaattcccat
aatccccaaaattttcagctacaacagcatat
